# Notes on Renal and Urinary Pathology

**Published:** 1880-09

**Authors:** 


NOTES ON RENAL AND URINARY PATHOLOGY.

1. "RENAL CALCULI; Their Causation, Character, Symptoms, Treatment and Prevention." By ROBERT L. WEIER, M.D., etc. (*N. Y. Medical Journal*, August, 1880.)

Dr. Weier presents one item of interest in this lecture, namely, the presence of the "submorphous" form of oxalate of lime, and of the urates in the nuclei of urinary calculi, and the fact that "these submorphous crystals are deposited under certain special conditions, viz.: the presence of a colloid material, which may consist of mucous or some other organic substance, but * * * this is an essential element in the formation of stone." As to the prevention of renal calculi, the essential thing, according to Dr. Weir's philosophy, is to secure and maintain proper dilution of the urine.

2. "A CLINIC ON CASES OF NEPHRITIS," etc. By FRANCIS DELAFIELD, M. D., Professor, etc. (Detroit *Lancet*, August, 1880.)

Dr. Delafield presents a case of nephritis in which he regarded it as impossible to make a certain differential diagnosis. The patient was a "young man," who had been "sick for a year, and the prominent features of his illness have been the existence of a moderate amount of dropsy, which is now diminishing, and a certain amount of anæmia." The diagnostic problem was to decide as between "chronic diffuse nephritis" and "chronic parenchymatous nephritis," which, as the case then stood, was regarded as impossible. Dr. Delafield, however, was inclined to regard the case as one of diffuse nephritis, because "the small number of casts in proportion to the albumen would rather point in this direction. When parenchymatous nephritis has lasted for some time, the proportion is rather the other way: the patients have albumen in the urine, but they also have casts, and casts in considerable numbers, and the casts are apt to be a more constant feature than albumen; whereas, with diffuse nephritis, the conditions are the other way: the albumen is apt to be more constant and in larger quantity than the casts." (We believe, however, that the "*architecture*" of the casts is a more reliable guide than that indicated by Dr. Delafield, although that is by no means without value.)

3. "ADDISON'S DISEASE." By THOS. F. ROCHESTER, M.D., Professor, etc., University of Buffalo. (*Buffalo Med. and Surg. Journal*, August, 1880.)

Prof. Rochester relates the history and presents the results of post mortem examination of three cases of morbus Addisonii. In neither case was the morbid anatomy by any means confined to the supra renal capsules; wherefore Prof. Rochester concludes with Martineau, "that the malady of Addison should not be considered as a pathological entity," and that "the question of individual pathological identity still remains open." (We believe that this question is no longer an "open" one; exactly *what* Addison's disease is must be regarded as an open question, but it is certainly something more than disease of the supra renal bodies.)

4. "INDICAN IN HEALTH AND DISEASE." By H. N. HEINEMAN, M.D., Assistant to Laboratory of the Alumni Association of the College of Physicians and Surgeons, New York. (*Archives of Medicine*, August, 1880.)

Dr. Heineman presents a very valuable paper on the physiological and pathological relations of indican. In an analysis of 149 cases of normal urine by Hoppe-Seyler and himself, indican was present in 116. In diseased states, indican was found present as follows:

*Circulatory system.*—Of five cases of cardiac disease three exhibited a normal amount, two a moderate and marked increase respectively. In one case of aneurism, indican was absent.

*Respiratory system.*—Of six cases of pleurisy, pneumonia and empyema, two had a normal quantity, two a moderate and two a marked increase. Senator states that in pneumonia and pleurisy he has found a moderate increase.

Of thirteen cases of acute and chronic phthisis two showed marked, three moderate increase, five a normal or slight amount and three absence. Hennige states that the condition of the intestine, whether diarrhœa be present or not, determines the increase in phthisis. Senator, with whom I agree, states with or without diarrhœa there is a moderate or marked increase.

*Digestive system.*—In three cases of stomach dyspepsia and one of dilatation there was marked increase. In a few cases of ulcer of the stomach, Senator has met with moderate increase. In numerous cases (number not stated) of gastro-duodenitis, Hennige found very marked increase. In one case I have found absence of indican and in three marked increase. In five cases of diarrhœa, there was marked increase in all. Of three cases of dysentery, two exhibited a slight amount and one none; of four cases of chronic constipation, two exhibited absence, two marked increase. Senator, and with him, Hennige, Jaffe, Edlefsen and DeVries, find that in simple constipation from atony, without invagination, only a slight amount of indican is present. The result in two cases met with by me is at variance with this. In one case of intussusception the increase was very marked. In seven cases of peritonitis there was marked increase, and this was greatest in the acute cases, and in those which were general rather than those that were localized. In four cases of intestinal hæmorrhage there was marked increase. In one case of cholera morbus there was marked increase. Senator, Wyss and Gubler have found marked increase in this, and in cases of Asiatic cholera.

*Liver.*—Of nine cases of cirrhosis, two only exhibited a moderate and marked increase respectively, two were normal, and in five there was none.

*Genito-urinary system: Kidneys.*—Of sixteen cases of Bright's disease, ten presented a marked and four a moderate increase, in two indican was absent.

Senator states that he has found no increase in any except cases of atrophied granular kidney. Heller, Scherer and Virchow had previously reported increase in indican in kidney lesions other than the one just mentioned. My own cases confirm this latter view. Of seven cases of Addison's disease, three had moderate and four marked increase.

*Bladder and Urethra.*—Of twelve cases in which pus was found in the urine in connection with urethral stricture, cystitis and gonorrhœa, seven gave moderate and one marked increase, three normal or slight amount, and one none.

*Uterus and Ovaries.*—One case of uterine disease had marked increase; one case of pueperal fever, normal or slight amount; in two cases of ovarian tumor none, and in three cases of pregnancy one had marked and two moderate increase.

*Osseous system.*—Of nine cases of arthritis, caries and necrosis, four had moderate increase, three normal or slight amount and four none. Of six cases of suppuration and cellulitis unconnected with bone disease, three had moderate increase, two normal amount and one none.

*Nervous system.*—Indican was absent in one case of apoplexia meningea; markedly increased in one of prog. muscular atrophy, in one case of insanity and epilepsy. In three cases of paraplegia, two had marked increase and one normal amount. In one case of sciatica the quantity was normal. In two cases of cerebral tumor and one of hypochondriasis and nocturnal emissions there was absence. Several authorities claim that spinal irritation is generally accompanied by an increase of indican.

*General diseases.*—Of five cases of alcoholism two had moderate and one marked increase and two were normal.

Of twenty cases of rheumatism eight of ten of acute disease were normal, one had marked increase and one none. In four of ten chronic cases the quantity was normal or slight, markedly increased in four and moderately in two.

Of nineteen cases of malaria six were normal, five had moderate and one marked increase and seven had none. Of six cases of typhoid fever one had moderate and five marked increase. Hennige has found the increase in typhoid fever dependent upon the presence of diarrhœa. Senator and I have found it independent.

In one case of convalescence from yellow fever the quantity was normal. Of ten cases of constitutional syphilis four had moderate and one marked increase, two were normal, three had none.

Of six cases of chlorosis three had moderate increase and three normal or slight amount. There was a normal or slight quantity in two cases of Werlhof's disease, marked increase in one case of progressive pernicious anæmia and absence in one case of leucocythæmia. In three cases of trichinosis and two of lead, poisoning there was marked increase. In one of chronic arsenical poisoning, absence.

In twenty-one cases of carcinoma interna there was very marked increase in nineteen and absence in two.

In three cases of abdominal lympho-sarcoma and in one case of osteo-sarcoma there was marked increase in all.

Dr. Hineman arrives at the following conclusions:

*Conclusions.*—Indican is only exceptionally absent in health. It may vary in quantity in the healthy individual, generally being small in amount, but occasionally as marked as in disease.

Certain diseased conditions tend to produce a decided increase.

The most marked increase is obtained in those diseases which affect the alimentary canal, and more especially the small intestine, whether they be local or general diseases with local lesion. Among these are dyspepsia, gastro-duodenitis, chronic constipation, intussusception, diarrhœa, peritonitis, cholera, lead poisoning and typhoid fever.

In diseases causing inanition, as phthisis and other prolonged suppurative diseases, as caries and necrosis, marked increase is found.

Among the diseases producing altered blood state, some, such as malaria, syphilis, rheumatism and alcoholism, progressive anæmia and chlorosis, although *always* causing the most profound blood-changes, *do not always* effect a decided increase. This is a singular exception, to which Senator has called attention, but the investigations are not as yet sufficiently numerous to draw conclusions.

Certain nervous disorders, as insanity, epilepsy, Addison's disease, progressive muscular atrophy and paraplegia, cause marked increase.

Disordered function of the kidneys, as in chronic Bright's disease, caused marked increase.

Internal malignant tumors, as carcinomata and sarcomata, cause increase most markedly and constantly of all.

The explanation of the cause of the increase in various diseases is not as yet adequate to satisfy all cases.

The experiment of Jaffe of ligating the small intestine, by which the amount of indican excreted was increased, demonstrates that conditions retarding peristalsis and favoring the absorption of indol, cause an increase of indican. This reasonably explains the result in cases of peritonitis, constipation and similar disorders.

How, satisfactorily, to account for the result in other diseases must, I regret to state, still remain unanswered.

The theory of Hennige that other diseases act through the nervous system and produce a change in the nature of the pancreatic secretions, and thus cause an increase, is effectually answered by Senator. He calls attention to the fact that indol. the mother substance of indican, is a product of decomposition. Furthermore, Brieger has shown that indol is produced without the presence of pancreatic juices.

Possibly in changes in the circulating fluid, rather than in local disturbances of the system, we may find the reason for the increase in the remaining diseases.

5. "FATAL CASE OF AGUE WITH DELIRIUM AND COMA DURING THE PAROXYSMS DUE TO CIRRHOTIC KIDNEY. By WM. RUSSELL, M.B., ED., late House Physician and Pathologist to the General Hospital, Wolverhampton. (*Edinburgh Medical Journal*, August, 1880.)

The following case came under my notice when in Perth as *locum tenens* for Dr. Stirling, and is sufficiently interesting and uncommon to warrant a few notes of it being recorded :

Mrs. I., aged sixty-nine, was seen by me on the night of 4th October. She had traveled from Glasgow that day, and was supposed to be in fair health when she left; she had, however, been under treatment for what was belived to be a hysterical condition. Since her death I have received particulars of the case from her Glasgow medical attendant. She had returned from America some three weeks before I saw her, and on the passage home had shown strange mental symptoms. During the first week or two of her stay in Glasgow she was said to have a regular nocturnal "shivering fit," after which she used to wander about in her night-gown. Some time after this she was seen by her medical attendant in one of her "shivering fits," the seizures at this time taking place in the forenoon instead of during the night, when he found her pulse quiet, her surface temperature feeling normal, and, when interested in conversation, that the shivering ceased. He considered the possibility of the case being ague, but was satisfied to the contrary. The urine was examined, and contained no albumen.

When I saw her, she complained of pain and confusion in her head, of frequent defecation, and of only passing a few scybala at a time. She was restless, had a dreamy and somewhat wearied expression, answered questions rationally, but did not recognize the friend she traveled to Perth to see. I ordered some bromide of potassium, and she passed a comfortable night. On the morning of the 5th she seemed much better and more collected, and the bowels had been freely moved by castor oil. In the morning I was summoned to see her, and found her tossing about in bed, occasionally groaning and moaning loud enough to be heard in the houses on the opposite side of the street. She was quite unconscious, and passed urine and fæces into the bed. The pupils responded to light. Her pulse was beating from 130 to 140, the heart's action being very tumultuous and thumping; the temperature in the axilla was 105°. Large doses of chloral hydrate and bromide of potassium quieted her. In the evening Dr. Bramwell kindly saw her in consultation with me. By that time her temperature had fallen to 102°, and the pulse to about 100, and she was regaining consciousness. From the existence of subcrepitant râles all over the left chest, it was suggested she had been knocked down by pneumonia, especially as we ascertained she had had a "shivering fit" during the afternoon. She was put on 5 grs. of quinine every six hours. On the morning of the 6th she seemed quite well again, her temperature being normal, and her pulse 84. During the day Dr. Bramwell saw her with me, when we congratulated ourselves and her on the marked improvement in her condition. In the evening I was again called to see her, and found her exhibiting the same train of nervous symptoms as on the previous evening. This annihilated our pneumonia theory. The temperature was 103°.5, and the pulse about 130. On investigation I found she had had another rigor, that with the onset of the rigor she became restless, and from that time the nervous symptoms had increased until she became unconscious, tossing about and moaning loudly from time to time. The initial symptoms so resembled ague that I examined the spleen and found it greatly enlarged. This new light on a case that had hitherto been obscure was satisfactory; but I was not satisfied that this was sufficient to explain the extreme severity of the cerebral symptoms, which seemed as out of keeping with an ague, with a temperature of only about 104°, as I believed them to be inexplicable by a pneumonia in a person of her years. I purposed to examine the urine when she became conscious, as I had no doubt she would the following morning. The coma, however, deepened, and she died that night.

By the kind influence of her Glasgow medical attendant the friends gave permission for a post-mortem examination. Dr. Gillespie, house surgeon to the Perth Infirmary, kindly assisted me. The *dura mater* was adherent to the calvarium, and had to be removed with it. The *brain* was hyperæmic, as shown by the size and number of the *puncta cruenta*, but was otherwise normal. The *heart* was large and fatty. The *left lung* was bound by old adhesions, and both it and its fellow were œdematous, but neither presented a trace of pneumonia. The *spleen* was much enlarged, measuring about 6 in. by 4 in., dark and pulpy. The *liver* was normal. The *kidneys* were small and markedly cirrhotic, no part of their capsules stripping off without lacerating their substance.

Although we tolerably frequently have the opportunity of seeing cases of ague in this country, the above case was at first peculiarly difficult, from the meagre and misleading history we possessed at the time; but the point of real interest in the case was the prominence and severity of the nervous symptoms, which at first gave one the idea of being due to active cerebral mischief, and was only given up when the first lull came; then the possibility of their being due to acute pneumonia was contemplated; and, at last, when the true nature of the seizures was evident, and with the light of the autopsy, we see the important *rôle* played by the state of the kidneys in producing cerebral irritation, manifested by restlessness and delirium during the course of the ague paroxysm, and afterwads a coma, which was ultimately fatal. I have seen patients suffering from other diseases in which a cirrhotic condition of kidney intensified the cerebral symptoms, and it is a complication I have come to regard tentatively as explaining the severity of the nervous symptoms in many cases in which we are apt to give the more prominent disease the onus of their severity, when in reality the more evident affection is quite insufficient to explain such intensity.

The relation between this case and what is described as malignant or pernicious ague is of some interest; for, even in the East, malignant ague seems to be very uncommon, and, when it does occur, kills early, presumably by the intensity of the poison, so that I should be inclined to question the possibility of our seeing such cases at home.

While we have no doubt that the pathological conditions of the kidneys plays an important part in the causation of the symptoms present in Mr. Black's case, it seems to us by no means certain that the "delirium and coma" are wholly due to "cirrhotic kidney," for the reason that the post mortem revealed quite too many other and important pathological lesions.

